# Social Decision Making and Suicidal Behavior in Late-Life Depression

**DOI:** 10.1093/geroni/igaa057.2116

**Published:** 2020-12-16

**Authors:** Katalin Szanto

**Affiliations:** University of Pittsburgh, Pittsburgh, Pennsylvania, United States

## Abstract

Social motivations to engage in suicide in late life frequently include interpersonal problems and escape from perceived defeat. To describe decision making patterns that may contribute to the catastrophic decision to take one’s life, we used behavioral experiments and assessed cognitive abilities and personality traits. We found that neuroticism, low extraversion, and low conscientiousness characterize older adults who contemplate suicide and those with low-lethality suicide attempts. Employing a novel version of the Ultimatum Game, we measured empathy’s moderating response to social conflict. We found that older suicide attempters were less influenced by empathy scenarios, indicating that a failure to integrate others’ emotions into decisions may undermine social deterrents to suicide. To simulate social status loss, we used a newly developed, competitive task (rigged toward primarily losing outcomes) paired with performance ranking. We found that suicide attempters, especially those with narcissistic traits, engaged in more excessive compensatory behaviors than older non-attempters.

